# Abnormal social behavior, hyperactivity, impaired remote spatial memory, and increased D1-mediated dopaminergic signaling in neuronal nitric oxide synthase knockout mice

**DOI:** 10.1186/1756-6606-2-19

**Published:** 2009-06-18

**Authors:** Koichi Tanda, Akinori Nishi, Naoki Matsuo, Kazuo Nakanishi, Nobuyuki Yamasaki, Tohru Sugimoto, Keiko Toyama, Keizo Takao, Tsuyoshi Miyakawa

**Affiliations:** 1 Genetic Engineering and Functional Genomics Group, Horizontal Medical Research Organization, Kyoto University Graduate School of Medicine, Kyoto, Japan; 2 Department of Pediatrics, Kyoto Prefectural University of Medicine, Kyoto, Japan; 3 Department of Pharmacology, Kurume University School of Medicine, Kurume, Japan; 4 Japan Science and Technology Agency (JST), Core Research for Evolutional Science and Technology (CREST), Kawaguchi, Japan; 5 Division of Systems Medical Science, Institute for Comprehensive Medical Science, Fujita Health University, Toyoake, Japan; 6 Japan Science and Technology Agency (JST), Institute for Bioinformatics Research and Development (BIRD), Kawaguchi, Japan

## Abstract

**Background:**

Neuronal nitric oxide synthase (nNOS) is involved in the regulation of a diverse population of intracellular messenger systems in the brain. In humans, abnormal NOS/nitric oxide metabolism is suggested to contribute to the pathogenesis and pathophysiology of some neuropsychiatric disorders, such as schizophrenia and bipolar disorder. Mice with targeted disruption of the nNOS gene exhibit abnormal behaviors. Here, we subjected nNOS knockout (KO) mice to a battery of behavioral tests to further investigate the role of nNOS in neuropsychiatric functions. We also examined the role of nNOS in dopamine/DARPP-32 signaling in striatal slices from nNOS KO mice and the effects of the administration of a dopamine D1 receptor agonist on behavior in nNOS KO mice.

**Results:**

nNOS KO mice showed hyperlocomotor activity in a novel environment, increased social interaction in their home cage, decreased depression-related behavior, and impaired spatial memory retention. In striatal slices from nNOS KO mice, the effects of a dopamine D1 receptor agonist, SKF81297, on the phosphorylation of DARPP-32 and AMPA receptor subunit GluR1 at protein kinase A sites were enhanced. Consistent with the biochemical results, intraperitoneal injection of a low dose of SKF81297 significantly decreased prepulse inhibition in nNOS KO mice, but not in wild-type mice.

**Conclusion:**

These findings indicate that nNOS KO upregulates dopamine D1 receptor signaling, and induces abnormal social behavior, hyperactivity and impaired remote spatial memory. nNOS KO mice may serve as a unique animal model of psychiatric disorders.

## Background

Establishing animal models of psychiatric disorders by utilizing genetically engineered mice is essential for investigating the pathogenesis, pathophysiology, and treatment of the disorders [1-5]. Previously, we reported that forebrain-specific calcineurin (also called protein phosphatase 2B) knockout (KO) mice have severe working/episodic-like memory deficits [6] and exhibit a spectrum of abnormal behaviors similar to those of schizophrenic patients [7]. In addition, we identified the *PPP3CC *gene, which encodes the calcineurin gamma subunit, as a potential schizophrenia susceptibility gene [8]. These studies demonstrated the usefulness of a comprehensive behavioral test battery for genetically engineered mice to efficiently evaluate a mouse model of human psychiatric disorders. Thus, we have applied this approach to test various strains of mice bearing mutations of genes encoding molecules involved in calcineurin signaling pathways or calcineurin-related neural mechanisms [5,9,10]. Here we focused on neuronal nitric oxide synthase (nNOS), one of the calcineurin substrates in the nervous system [11,12].

Nitric oxide (NO) is a highly diffusible gas that acts as an endogenous messenger molecule in various tissues. In the brain, NO has a variety of important roles, including regulation of neurotransmission, synaptic plasticity, gene expression, and neurotoxicity [13-15]. NO is enzymatically synthesized from L-arginine by nitric oxide synthase (NOS). In the mammalian nervous system, NO is primarily produced by nNOS, an isoform predominantly expressed in the brain among three NOS isoforms [14]. nNOS is expressed in a discrete population of neurons in the hippocampus, cortex, striatum, cerebellum, olfactory bulb, and brain stem [16,17]. nNOS catalytic activity is regulated by the phosphorylation state of the enzyme. The phosphorylation of nNOS by protein kinase C (PKC) and Ca^2+^/calmodulin-dependent kinases inhibits nNOS activity [18,19], whereas dephosphorylation by calcineurin activates nNOS [20]. Direct binding of nNOS to PSD-95 protein induces nNOS to localize at a postsynaptic density in the vicinity of NMDA receptors, allowing for an efficient and specific activation of nNOS in response to a glutamate-induced Ca^2+ ^influx [21,22].

The *in vivo *function of nNOS has been examined using mice with targeted disruption of the nNOS gene [23]. These mice are viable and exhibit a grossly normal appearance, but their aggressive behavior [24], nocturnal motor coordination [25], and cognitive performance [26] are somewhat abnormal. In humans, abnormal nNOS/NO metabolism is suggested to contribute to the pathogenesis and pathophysiology of some neuropsychiatric disorders. In postmortem brain from patients with schizophrenia, the total number of nNOS-containing neurons in the hypothalamus is smaller [27] and nNOS-positive striatal interneurons are decreased [28] compared to normal cases. Regulatory polymorphisms of nNOS contribute to the genetic risk for schizophrenia [29] and the nNOS gene is associated with schizophrenia among Ashkenazi Jewish case-parent trios [30]. Recently, Walsh et al. reported more microdeletions and microduplications in the genome of schizophrenia patients compared to control samples [31]. The microdeletions and microduplications in cases disproportionately disrupted genes involved in some signaling networks, including NO signaling pathways [31]. Among the several pathways and processes overrepresented by disrupted genes in schizophrenia cases, NO signaling pathways were the most statistically reliable [31]. In addition, transcription of nitric oxide synthase 1 (neuronal) adaptor protein (NOS1AP) that is also termed CAPON, was upregulated both in schizophrenia and bipolar disorder [32]. Binding of NOS1AP to nNOS results in a reduction of NMDA receptor/NOS complexes, leading to decreased NMDA receptor-gated calcium influx and a catalytically inactive nitric oxide synthase [33]. In agreement, genetic association study revealed that single nucleotide polymorphisms (SNP) in NOS1AP were associated with schizophrenia [34]. The variant with the SNP altered the expression of the gene by enhancing transcription factor binding [34].

The interaction between glutamatergic and dopaminergic pathways is crucial for cognitive and motor functions, and both signal transduction pathways are major contributing factors in schizophrenia pathogenesis [35]. DARPP-32, which is a 32-kDa dopamine- and cyclic adenosine monophosphate (cAMP)-regulated phosphoprotein, is a critical signal transduction molecule that integrates glutamatergic and dopaminergic pathways in medium spiny neurons in the neostriatum [36,37]. Dopamine, acting through D1 receptors, activates cAMP-dependent protein kinase (PKA), resulting in the phosphorylation of DARPP-32 at Thr34 [36,38]. Phosphorylated DARPP-32 is a potent inhibitor of protein phosphatase-1 [39], and thereby controls the phosphorylation state and activity of many downstream molecules, such as NMDA receptors, AMPA receptors, voltage-dependent Na^+ ^channels, and Ca^2+ ^channels [40]. On the other hand, glutamate activates calcineurin, resulting in the dephosphorylation and inactivation of DARPP-32 [41-43]. Glutamate also activates the nNOS/NO/cyclic guanine monophosphate (cGMP)/protein kinase G (PKG) signaling cascade, leading to the phosphorylation of DARPP-32 at Thr34 [44,45], as DARPP-32 at Thr34 is an excellent substrate for PKG as well as for PKA [39]. Moreover, NO may also inhibit dopamine uptake [46]. Thus, nNOS/NO signaling is under the control of glutamate, and has an important role in the regulation of dopaminergic/DARPP-32 signaling.

To assess the possible utility of nNOS KO mice as an animal model of psychiatric disorders, we subjected them to a comprehensive behavioral test battery [5,9]. nNOS KO mice showed hyperlocomotor activity in a novel environment, increased social interaction in their home cage, decreased depression-related behavior, and impaired spatial memory retention. We also examined a possible role for nNOS in dopaminergic signaling and revealed an upregulation of the dopamine-signaling cascade in neostriatal slices from nNOS KO mice. The augmenting effect of D1 agonist administration on sensorimotor gating in prepulse inhibition (PPI) tests is consistent with an upregulation of the dopamine pathway in nNOS KO mice.

## Methods

### Animals and Experimental Design

nNOS KO (Strain Name: B6;129S4-*Nos1*^*tm1hlh*^/J, Stock Number: 002633) mice were obtained from Jackson Laboratories (Bar Harbor, ME) [23]. They were backcrossed for five generations onto a C57BL/6J background. We could not backcross for any more generations due to the infertility of nNOS KO mice [47]. Genetic testing of two nNOS KO mice confirmed that an average of 94.5% of the markers corresponded to C57BL/6J (Genetic Testing Services; Central Institute for Experimental Animals, Tokyo). nNOS KO mice and wild-type control littermates were obtained by breeding heterozygote mice. All behavioral tests were carried out with male mice that were at least 7 weeks old at the start of testing. Raw data of the behavioral test, the date on which each experiment was done, and the age of the mice at the time of the experiment are shown in the mouse phenotype database . Mice were group housed (2–4 mice per cage) in a room with a 12 hr light/dark cycle (lights on at 7:00 a.m.) with access to food and water *ad libitum*. Room temperature was kept at 23 ± 2°C. Behavioral testing was performed between 9:00 a.m. and 6:00 p.m. After the tests, all apparatus was cleaned with diluted sodium hypochlorite solution to prevent a bias due to olfactory cues. We prepared four independent groups of mice for behavioral tests. One group consisted of the equivalent number of nNOS KO mice and wild-type control littermates. Experiments were done in the following sequences; the first group: the general health and neurological screen including wire hang test (GHNS), light/dark transition (LD), open field (OF), elevated plus maze (EP), hot plate (HP), one-chamber social interaction test (SI), rotarod (RR), startle response/prepulse inhibition test (PPI), Morris water maze, social interaction test in home cage (HC-SI) and latent inhibition test; the second group: GHNS, LD, OF, EP, HP, SI, RR, PPI, Porsolt forced swim test (PS), HC-SI and eight-arm radial maze; the third group: GHNS, LD, OF, EP, SI, Crawley's sociability and preference for social novelty test (CSI) and PPI with drug; the forth group: GHNS, LD, OF, EP, SI, CSI and PPI with drug. Each behavioral test was separated from each other at least by 1 day. There were no significant interactions between genotype and group in the results presented in any figures (ANOVA, p > 0.05). All behavioral testing procedures were approved by the Animal Care and Use Committee of Kyoto University Graduate School of Medicine.

### Behavioral testing

#### Open field test

Locomotor activity was measured using an open field test. Each mouse was placed in the corner of the open field apparatus (40 × 40 × 30 cm; Accuscan Instruments, Columbus, OH). The chamber of the test was illuminated at 100 lux. Total distance traveled (in cm), vertical activity (rearing measured by counting the number of photobeam interruptions), time spent in the center area, and beam-break counts for stereotyped behaviors were recorded. Data were collected for 120 min.

#### Light/dark transition test

A light/dark transition test was conducted as previously described [48]. The apparatus used for the light/dark transition test comprised a cage (21 × 42 × 25 cm) divided into two sections of equal size by a partition with a door (Ohara & Co., Tokyo). One chamber was brightly illuminated (390 lux), whereas the other chamber was dark (2 lux). Mice were placed into the dark side and allowed to move freely between the two chambers with the door open for 10 min. The total number of transitions, latency to first enter the lit chamber, distance traveled, and time spent in each chamber were recorded by Image LD4 software (see 'Data analysis').

#### Elevated plus maze test

An elevated plus maze test was conducted as previously described [49]. The elevated plus-maze consisted of two open arms (25 × 5 cm) and two enclosed arms of the same size with 15-cm high transparent walls. The arms and central square were made of white plastic plates and were elevated 55 cm above the floor. To minimize the likelihood of animals falling from the apparatus, 3-mm high Plexiglas walls surrounded the sides of the open arms. Arms of the same type were located opposite from each other. Each mouse was placed in the central square of the maze (5 × 5 cm), facing one of the closed arms. Mouse behavior was recorded during a 10-min test period. The number of entries into an arm, and the time spent in the open and enclosed arms were recorded. Percentage of entries into open arms, time spent in open arms (s), number of total entries, and total distance traveled (cm) were analyzed. Data acquisition and analysis were performed automatically, using Image EP software (see 'Data analysis').

#### Social interaction test in a novel environment (one-chamber social interaction test)

In the social interaction test, two mice of identical genotypes that were previously housed in different cages were placed in a box together (40 × 40 × 30 cm) and allowed to explore freely for 10 min. Social behavior was monitored with a CCD camera connected to a Macintosh computer. Analysis was performed automatically using Image SI software (see 'Data analysis'). The total number of contacts, total duration of active contacts, total contact duration, mean duration per contact, and total distance traveled were measured. The active contact was defined as follows. Images were captured at 1 frame per second, and distance traveled between two successive frames was calculated for each mouse. If the two mice contacted each other and the distance traveled by either mouse was longer than 2 cm, the behavior was considered as 'active contact'.

#### Social interaction test in home cage

Social interaction monitoring in the home cage was conducted as previously described [7]. The system comprised the home cage (29 × 18 × 12 cm) and a filtered cage top, separated by a 13-cm-high metal stand containing an infrared video camera attached at the top of the stand. Two mice of the same genotype that had been housed separately were placed together in a home cage. Their social behavior was then monitored for 1 week. Output from the video camera was fed into a Macintosh computer. Images from each cage were captured at a rate of one frame per second. Social interaction was measured by counting the number of particles detected in each frame: two particles indicated that the mice were not in contact with each other; and one particle (i.e., the tracking software could not distinguish two separate bodies) indicated contact between the two mice. We also measured locomotor activity during these experiments by quantifying the number of pixels that changed between each pair of successive frames. Analysis was performed automatically using Image HA software (see 'Data analysis').

#### Crawley's sociability and preference for social novelty test

The test for sociability and preference for social novelty was conducted as previously described [50,51]. The apparatus comprised a rectangular, three-chambered box and a lid containing an infrared video camera (Ohara & Co.). Each chamber was 20 × 40 × 22 cm and the dividing walls were made from clear Plexiglas, with small square openings (5 × 3 cm) allowing access into each chamber. An unfamiliar C57BL/6J male (stranger 1) that had no prior contact with the subject mouse was placed in one of the side chambers. The placement of stranger 1 in the left or right side chambers was systematically alternated between trials. The stranger mouse was enclosed in a small, circular wire cage that allowed nose contact between the bars, but prevented fighting. The cage was 11 cm high, with a bottom diameter of 9 cm and bars spaced 0.5 cm apart. The subject mouse was first placed in the middle chamber and allowed to explore the entire social test box for 10-min. The amount of time spent within a 5-cm distance of the wire cage and in each chamber. At the end of the first 10 min, each mouse was tested in a second 10-min session to quantitate social preference for a new stranger. A second, unfamiliar mouse was placed in the chamber that had been empty during the first 10-min session. This second stranger was enclosed in an identical small wire cage. The test mouse had a choice between the first, already-investigated unfamiliar mouse (stranger 1), and the novel unfamiliar mouse (stranger 2). As described above, the amount of time spent within a 5-cm distance of each wire cage and in each chamber during the second 10-min session was recorded. The stranger mice used in this experiment were 8 to 12-week-old C57BL/6J male mice, not littermates. Analysis was performed automatically using Image CSI software (see 'Data analysis').

#### Startle response/PPI test

A startle reflex measurement system was used (Ohara & Co.). A test session began by placing a mouse in a Plexiglas cylinder where it was left undisturbed for 10 min. The duration of white noise that was used as the startle stimulus was 40 ms for all trial types. The startle response was recorded for 140 ms (measuring the response every 1 ms) starting with the onset of the prepulse stimulus. The background noise level in each chamber was 70 dB. The peak startle amplitude recorded during the 140-ms sampling window was used as the dependent variable. A test session consisted of 6 trial types (i.e., two types for startle stimulus-only trials, and four types for PPI trials). The intensity of the startle stimulus was 110 or 120 dB. The prepulse sound was presented 100 ms before the startle stimulus, and its intensity was 74 or 78 dB. Four combinations of prepulse and startle stimuli were employed (74–110 dB, 78–110 dB, 74–120 dB, and 78–120 dB). Six blocks of the 6 trial types were presented in a pseudorandom order such that each trial type was presented once within a block. The average inter-trial interval was 15 s (range, 10–20 s).

In the PPI test with drug treatment, nNOS KO and wild-type mice were assigned to receive either the selective D1 receptor agonist SKF81297 (1 mg/kg) or saline (balanced for genotype, startle chamber assignment, and treatment) and were tested for PPI 20 min later. SKF81297 was dissolved in saline, and both SKF81297 and saline were administered intraperitoneally in an injection volume of 10 ml/kg.

#### Porsolt forced swim test

The Porsolt forced swim test apparatus consisted of four Plexiglas cylinders (20 cm high × 10 cm diameter). A nontransparent panel separated the cylinders to prevent the mice from seeing each other (Ohara & Co.). The cylinders were filled with water (23°C) up to a height of 7.5 cm. Mice were placed into the cylinders, and their behavior was recorded over a 10-min test period. Retention tests were administered 24 hours after training (trial 2) and 1 week after the first test (trial 3). Data acquisition and analysis were performed automatically, using Image PS software (see 'Data Analysis').

#### Eight-arm radial maze test

The eight-arm radial maze test was conducted to assess spatial working memory in a manner similar to that described previously [10]. The floor of the maze was made of white Plexiglas and the wall (25 cm high) consisted of transparent Plexiglas. Each arm (9 × 40 cm) radiated from an octagonal central starting platform (12 cm/side) like the spokes of a wheel. A guillotine door separated each arm from the central starting platform. Identical food wells (1.4 cm deep and 1.4 cm in diameter) with pellet sensors were placed at the distal end of each arm. The pellets sensors automatically recorded pellet intake by the mice. The maze was elevated 75 cm above the floor and placed in a dimly lit room with several extra-maze cues. During the experiment, the maze was maintained in a constant orientation. One week before pretraining, animals were deprived of food until their body weight was reduced to 80% to 85% of the initial level. Pretraining started on the 8th day. Each mouse was placed in the central starting platform and allowed to explore and to consume food pellets scattered across the whole maze for a 30-min period (one session per mouse). After completing the initial pretraining, mice were given another pretraining session to take a pellet from each food well after being placed at the distal end of each arm. A trial was finished after the subject consumed the pellet. This was repeated 8 times, using 8 different arms, for each mouse. After these pretraining trials, maze acquisition trials were performed. All 8 arms were baited with food pellets. Mice were placed on the central platform and allowed to obtain all 8 pellets within 25 min. A trial was terminated immediately after all 8 pellets were consumed or 25 min had elapsed. An 'arm visit' was defined as traveling more than 5 cm into the arm from the edge of the central platform. The mice were confined in the center platform for 5 s after each arm choice. The animals were given one trial per day for 30 days (30 trials total). After the initial 30 trials, "delay trials" were conducted during which the mice were confined in the center platform for 30, 120, 300 s after performing 4 correct arm choices (2 trials for each delay). For each trial, the number of different arms chosen among the first 8 choices and the number of revisiting errors were automatically recorded. Data acquisition, control of guillotine doors, and data analysis were performed by Image RM software (see 'Data analysis').

#### Morris water maze test

The "hidden platform" version of the Morris water maze test was conducted to assess spatial reference memory, as described previously [52]. The apparatus consisted of a circular tank (40 cm high × 95 cm diameter) filled with water (up to 30 cm deep) maintained at room temperature (23 ± 2°C) that was made opaque with nontoxic white paint. The surface of the platform (8 × 8 cm) was 1 cm below the water surface. Four trials per day were conducted for 14 successive days with the same platform location. There were four possible locations for the platform. One of these platform positions was assigned to each mouse as the correct location during the training. Latency to reach the platform was recorded. When the distance between the mouse and the wall of the pool was less than 8 cm, the mouse was considered to be at the perimeter. On the 15th day, the platform was removed, and a 60-s probe trial was conducted (probe test A). Time spent in each quadrant was recorded during the probe trials. Four training trials were conducted immediately after the probe test A and another probe test (probe test B) was conducted 1 week after the initial probe test to evaluate memory retention. Data acquisition and analysis were performed using Image WM software (see 'Data analysis').

#### Data analysis

Behavioral data were obtained automatically by applications based on the public domain NIH Image program and Image J program and modified for each test by Tsuyoshi Miyakawa (available through Ohara & Co.). Statistical analysis was conducted using StatView (SAS Institute, Cary, NC). Data were analyzed by two-tailed t-test, two-way ANOVA, or two-way repeated measures ANOVA. Values in graphs are expressed as mean ± SEM.

### Analysis of protein phosphorylation in neostriatal slices

#### Preparation and incubation of neostriatal slices

Neostriatal slices were prepared from male wild-type and nNOS KO mice at 9 to 12 weeks of age. The mice were decapitated and the brains rapidly removed and placed in ice-cold, oxygenated Krebs-HCO_3_^- ^buffer (124 mM NaCl, 4 mM KCl, 26 mM NaHCO_3_, 1.5 mM CaCl_2_, 1.25 mM KH_2_PO_4_, 1.5 mM MgSO_4_, and 10 mM D-glucose, pH 7.4). Coronal slices (350 μm) were prepared using a vibrating blade microtome, VT1000S (Leica Microsystems, Nussloch, Germany). The striatum was dissected from the slices in ice-cold Krebs-HCO_3_^- ^buffer. Each slice was placed in a polypropylene incubation tube with 2 ml fresh Krebs-HCO_3_^- ^buffer containing adenosine deaminase (10 μg/ml). The slices were preincubated at 30°C under constant oxygenation with 95% O_2_/5% CO_2 _for 60 min. The buffer was replaced with fresh Krebs-HCO_3_^- ^buffer after 30 min of preincubation. Adenosine deaminase was included during the first 30 min of preincubation. Slices were treated with drugs as specified in each experiment. SKF81297 and glutamate were obtained from Sigma-Aldrich (St. Louis, MO). After drug treatment, slices were transferred to Eppendorf tubes, frozen on dry ice, and stored at -80°C until assayed.

#### Immunoblotting

Frozen tissue samples were sonicated in boiling 1% sodium dodecyl sulfate and boiled for an additional 10 min. Small aliquots of the homogenate were retained for protein determination by the BCA protein assay method (Pierce, Rockford, IL). Equal amounts of protein (100 μg) were separated by sodium dodecyl sulfate-polyacrylamide gel electrophoresis (10% polyacrylamide gels), and transferred to nitrocellulose membranes (0.2 μm) (Schleicher and Schuell, Keene, NH). The membranes were immunoblotted using phosphorylation state-specific antibodies: phospho-Thr34 DARPP-32 antibody (mAb-23; 1:750 dilution) [53]; phospho-Thr75 DARPP-32 antibody (1:5000 dilution) [54]; phospho-Ser845 GluR1 antibody (#06-773; 1:1000; Upstate Cell Signaling Solutions, Lake Placid, NY); phospho-ERK1/2 antibody (#9100; 1:2000 dilution; New England BioLabs, Beverly, MA); and phospho-Ser94 spinophilin antibody (RU499; 1:4000 dilution) [55]. The membrane was reblotted with DARPP-32, GluR1, ERK, or spinophilin antibody to determine the total amount of those proteins. None of the experimental manipulations used in the present study altered the total amount of DARPP-32, GluR1, ERK, or spinophilin.

The membrane was incubated with a goat anti-mouse or rabbit Alexa 680-linked IgG (1:5000 dilution; Molecular Probes, Eugene, OR) or a goat anti-mouse or rabbit IRDye™800-linked IgG (1:5000 dilution; Rockland, Gilbertsville, PA). Fluorescence at infrared wavelengths was detected by the Odyssey infrared imaging system (LI-COR, Lincoln, NE), and quantified using Odyssey software. In an individual experiment, samples from control and drug-treated slices were analyzed on the same immunoblot. For each experiment, values obtained for slices were calculated relative to values for the control slices from wild-type mice. Normalized data from multiple experiments were averaged and statistical analysis was performed as described in the figure legends.

## Results

### Increased locomotor activity in nNOS KO mice

**Figure 1:**
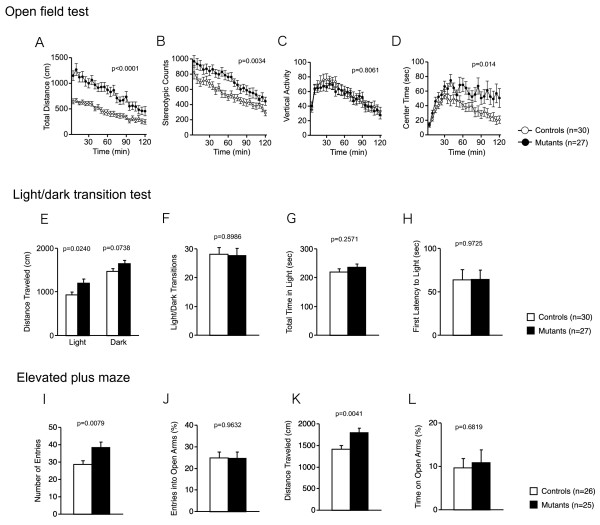
**Increased locomotor activity in nNOS KO mice**. (A-D) Open field test: total distance traveled (A), stereotypic behavior (B), vertical activity (C), and time spent in the center of the compartment (D) were recorded. (E-H) Light/dark transition test: distance traveled in the light/dark compartments (E), number of light/dark transitions (F), time spent in light compartment (G), and latency to enter the light compartment (H) were recorded. (I-L) Elevated plus maze test: number of arm entries (I), percentage of entry into open arms (J), distance traveled (K), and time spent on open arms (L) were recorded. The p values indicate genotype effect in two-way ANOVA.

Locomotor activity was examined in the open field test, elevated plus maze, light/dark transition test, and social interaction tests. nNOS KO mice showed a pronounced increase in locomotor activity in several different tests. Hyperactivity was consistently observed in all locomotor activity-related indices measured. Compared with wild-type control mice nNOS KO mice traveled significantly longer distances in the open field test [Fig 1A; genotype effect, F(1,55) = 26.395, p < 0.0001], in the lit compartment in the light/dark transition test [Fig 1E; genotype effect, F(1,55) = 5.392, p = 0.0240], and in the elevated plus maze test [Fig 1K; genotype effect, F(1,49) = 9.068, p = 0.0041]. The stereotypic counts in the open field test [Fig 1B; genotype effect, F(1,55) = 9.389, p = 0.0034], and the number of total arm entries in the elevated plus maze test [Fig 1I; genotype effect, F(1,49) = 7.671, p = 0.0079] were also significantly increased in nNOS KO mice. There was no significant difference in vertical activity of the open field test [Fig 1C; genotype effect, F(1,55) = 0.061, p = 0.8061].

We also assessed anxiety-like behaviors in nNOS KO mice. nNOS KO mice spent significantly more time in the center of the open field apparatus [Fig 1D; genotype effect, F(1,55) = 6.441, p = 0.014], which is considered to reflect reduced anxiety-like behavior. The lack of nNOS, however, did not significantly affect the following indices: the number of the light/dark transitions [Fig 1F; genotype effect, F(1,55) = 0.016, p = 0.8986] and the total time spent in the lit compartment [Fig 1G; genotype effect, F(1,55) = 1.312, p = 0.2571] and first latency to enter the light chamber [Fig 1H; genotype effect, F(1,55) = 0.001, p = 0.9725] in the light/dark transition test, the percentage of entries into the open arms [Fig 1J; genotype effect, F(1,49) = 0.002, p = 0.9632], and time on open arms [Fig 1L; genotype effect, F(1,49) = 0.170, p = 0.6819] in the elevated plus maze test, suggesting that anxiety-like behavior is not altered in nNOS KO mice. Thus, the lack of nNOS was not associated with consistent changes in anxiety-like behavior. Increased time in the center of open field in nNOS KO mice, however, might reflect hyperactivity.

**Figure 2:**
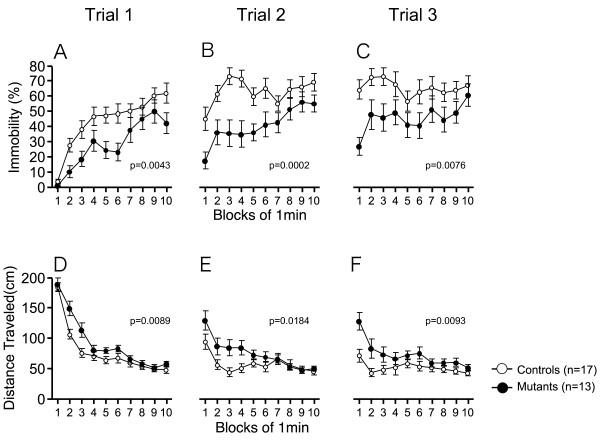
**Increased depression-related behavior in nNOS KO mice**. (A-C) Immobility and (D-F) distance traveled were recorded in the Porsolt forced swim test for three trials. The p values indicate genotype effect in two-way repeated measures ANOVA.

In the Porsolt forced swim test, increased immobility is interpreted as a form of learned helplessness that reflects depression-related behavior and/or increased stress-sensitivity [56]. nNOS KO mice spent significantly less time immobile than wild-type mice (Fig 2), demonstrating decreased depression-related behavior in nNOS KO mice. Moreover, nNOS KO mice traveled significantly longer distances, indicating that nNOS KO mice display a hyperactive phenotype under extremely stressful conditions.

### Abnormal social behavior of nNOS KO mice

**Figure 3:**
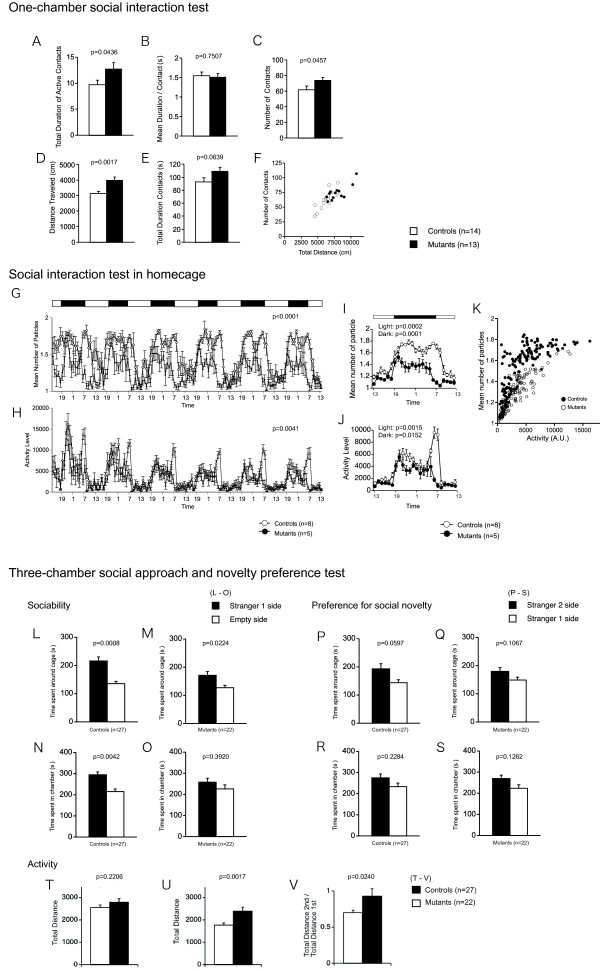
**Abnormal social behaviors in nNOS KO mice**. (A-F) Social interaction test in a novel environment (one-chamber social interaction test): total duration of active contacts (A), mean duration of each contact (B), number of contacts (C), total distance traveled (D), and total contact duration (E) were recorded. The relationship between distance traveled and number of contacts was plotted (F). The p values indicate genotype effect in two-way ANOVA (A-E). (G-I) Social interaction test in home cage: mean number of particles detected (G) and activity level (H) were recorded over 6 days. The averaged graph over 3 days of mean number of particles detected (I) and activity level (J). The relationship between activity level and number of particles was plotted (K). The p values indicate genotype effect in two-way repeated measures ANOVA (G-J). (L-V) Crawley's three-chamber social approach test. In the sociability test (L-V), time spent around the empty cage or the cage containing a stranger (L, M) and time spent in the chamber with an empty cage or cage containing a stranger (N, O) were recorded. In the preference for social novelty test (P-S), time spent around the cage containing a stranger (stranger 2) and the cage with a familiar mouse (stranger1) (P, Q) and time spent in the chamber with the cage containing a stranger (stranger 2) and the cage with a familiar mouse (stranger1) cage (R, S) were recorded. Distance traveled was recorded in both tests (T, U). The ratio of distance traveled in the sociability test to that traveled in the preference for social novelty test is shown (V). The p values indicate the difference between the cages (stranger side vs. empty side; L-O, stranger 2 side vs. stranger 1 side; P-S). The p values indicate genotype effect in two-way ANOVA (T-V).

During the social interaction test in a novel environment, nNOS KO mice traveled significantly longer distances [Fig 3D; genotype effect, F(1,25) = 12.430, p = 0.0017], and both the number of contacts and the total duration of active contacts between nNOS KO mice were greater than those of wild-type mice [Fig 3C; genotype effect, F(1,25) = 4.424, p = 0.0457; Fig 3A; genotype effect, F(1,25) = 4.518, p = 0.0436], suggesting that nNOS KO mice were hyperactive. The duration of contacts made by nNOS KO mice, however, tended to be longer than that of wild-type mice [Fig 3E; genotype effect, F(1.25) = 3.759, p = 0.0639] and mean duration per contact was not significantly different [Fig 3B; genotype effect, F(1,25) = 0.013, p = 0.7507]. The findings could also be the result of hyperactivity, as a previous meta-analysis data of over 1000 mice showed a high correlation between locomotor activity and the number of contacts or total duration of active contacts in the social interaction test (unpublished data). ANCOVA applied to the number of total contacts, with total distance as a covariate, indicated that the interaction between total distance and genotypes was not significant (p = 0.713) and the effect of genotype did not remain after including total distance traveled as the covariate in the ANCOVA (p = 0.1102). Therefore, the increased number of contacts of nNOS KO mice in the social interaction test in a novel environment may be due to hyperactivity (Fig 3F).

We monitored social interaction in the home cage under familiar conditions over a 6-day period. In the social interaction test in the home cage, time spent separated is usually increased when mice are active and decreased when mice are sleeping. nNOS KO mice spent significantly less time separated from each other than wild-type mice [Fig 3G; genotype effect, p < 0.0001] and locomotor activity was significantly lower in nNOS KO mice [Fig 3H; genotype effect, p = 0.0041]. These phenotypes were observed both in the light period [Fig 3I, J; genotype effect, mean number of particles, p = 0.0002; activity level, p = 0.0015] and in dark period [Fig 3I, J; genotype effect, mean number of particles, p = 0.0001; activity level, p = 0.0152]. Even at the same activity level, nNOS KO mice tended to remain separated compared to wild-type, indicating that the increased contact in nNOS KO mice was not due to hyperactivity (Fig 3K). ANCOVA applied to the mean number of particles detected, with activity level as a covariate, indicated a significant interaction between activity level and genotype (p = 0.0122) and the effect of genotype remained when activity was used as the covariate in the ANCOVA (p < 0.0001). Analysis of the relationship between activity level and the interaction between two mice indicated that nNOS KO mice showed an increased number of contacts at the same activity level compared to wild-type mice in their home cage. These findings indicate that nNOS deficiency induces an increase in social interaction in the familiar environment.

Crawley's three-chamber social approach test consists of sociability test and a social novelty preference test. These tests assess social interaction that is relatively independent of locomotor activity compared to the other social interaction tests, because the preference of the mice can be quantified based on the time spent around a wire cage containing a stranger mouse vs. an empty cage in the sociability test and stranger mouse vs. a familiar mouse [50]. In the sociability test, both nNOS KO mice and wild-type mice type demonstrated normal sociability [Fig 3L, M; time spent around cage, with stranger vs. empty; wild-type: t(26) = 3.804, p = 0.0008, nNOS KO: t(21) = 2.465, p = 0.0224, paired-t test]. In nNOS KO mice, however, social approach was decreased in the sociability test [time spent around the cage with the stranger, genotype effect, F(1,47)= 5.15, p = 0.0279]. Consistently, nNOS KO mice did not show a preference for the chamber with the stranger [Fig 3N, O; time spent in chambers (stranger 1 side vs. empty cage side); wild-type mice: t(26) = 3.134, p = 0.0042; nNOS KO mice: t(21) = 0.874, p = 0.3920, paired-t test]. The distance traveled in the sociability test was not significantly different between genotypes [Fig 3T; genotype effect, F(1,47) = 1.541, p = 0.2206]. In the social novelty preference test, wild-type mice tended to demonstrate a preference for novelty [Fig 3P; time spent around the cage containing stranger 1 vs. that containing stranger 2: t(26) = 1.969, p = 0.0597, paired-t test], whereas nNOS KO mice did not [Fig 3Q; time spent around the cage containing stranger 1 vs. that containing stranger 2: t(21) = 1.686, p = 0.1067, paired-t test]. A preference between chambers was not detected in either genotype [Fig 3R,S; time spent around cages, wild-type mice: t(26) = 1.234, p = 0.2284, nNOS KO mice: t(21) = 1.592, p = 0.1262, paired-t test]. Although there were no significant difference in the distance traveled in the sociability test (Figure 3T), nNOS KO mice traveled a greater distance in the novelty preference test [Fig 3U; genotype effect, F(1,47) = 11.094, p = 0.0017]. The ratio of distance traveled in the novelty preference test to the distance traveled in the sociability test was higher in nNOS KO mice [Fig 3V; genotype effect, F(1,47) = 5.439, p = 0.0240], suggesting that nNOS KO mice habituated less than wild-type mice.

### Performance deficits of nNOS KO mice in the memory tasks

**Figure 4:**
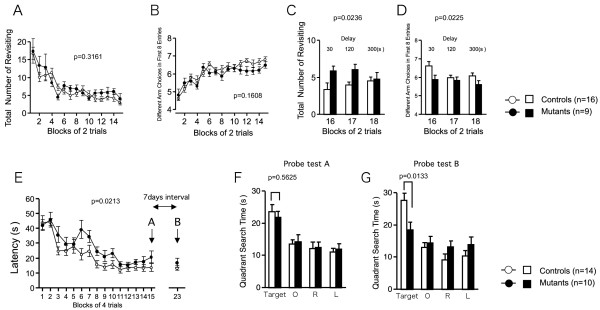
**Impaired remote spatial memory of nNOS KO mice**. (A-D) Eight-arm radial maze test: total number of arms revisited (A, C) and different arm choices among the first 8 entries (B, C) during training were recorded. During trials 32–36, a delay was applied after of the first 4 pellets were consumed (C, D). (E-G) Morris water maze test: latency to escape (E) was recorded during training session and probe tests. Probe tests were conducted 1 day (F) and 7 days after the last training trial (G). The p values indicate genotype effect in two-way repeated measures ANOVA (A-E) and genotype effect in two-way ANOVA (F, G).

In the eight-arm radial maze test (spatial working memory task), the number of revisiting errors, in which subjects returned to the arms that had been visited previously to retrieve a food pellet, was not significantly different between genotypes during trials without a delay [Fig 4A; genotype effect, F(1,23) = 1.050, p = 0.3161; genotype × trial interaction, F(14,322) = 0.990, p = 0.4636]. The number of different arm choices among the first 8 entries, which is considered a measure of working memory that is relatively independent of locomotor activity levels, and the total number of arm choices were not significantly different between genotypes [Fig 4B; genotype effect, F(1,23) = 2.100, p = 0.1608; genotype × trial interaction, F(14,322) = 1.325, p = 0.1905]. On the other hand, both the number of revisiting errors and the different arm choices among the first 8 entries were significantly greater in nNOS KO mice during trials with delays [Fig 4C; genotype effect, F(1,23) = 5.880, p = 0.0236; genotype × trial interaction, F(2,46) = 1.837, p = 0.1708, Fig 4D; genotype effect, F(1,23) = 5.985, p = 0.0225; genotype × trial interaction, F(2,46) = 1.350, p = 0.2693], suggesting that nNOS KO mice have mildly impaired working memory.

In the Morris water maze (spatial reference memory task), latency to locate the escape platform during hidden platform training (for 14 successive days) in nNOS KO mice was significantly greater than that in wild-type mice [Fig 4E; genotype effect, F(1,22) = 6.145, p = 0.0213; genotype × trial interaction, F(13,286) = 1.174, p = 0.2981]. Probe trials were performed on day 15 and day 23 (1 week after the last trial). Both nNOS KO mice and wild-type mice spent more time in the previously trained quadrant than in the three untrained quadrants. Time spent in the previously trained quadrant was not significantly different between genotypes on the probe trial of the day 15 [Fig 4F; genotype effect, F(1,22) = 0.346, p = 0.5625]. nNOS KO mice spent significantly less time in the trained quadrant than wild-type mice on day 23 [Fig 4G; genotype effect, F(1,22) = 7.636, p = 0.0133]. These data suggest that nNOS KO mice have impaired spatial remote memory.

In the Porsolt forced swim test, nNOS KO mice showed decreased depression-related behavior [Fig 2A–C; genotype effect, first trial: F(1,28) = 9.660, p = 0.0043, a day after the first trial: F(1,28) = 18.554, p = 0.0002, 7 days after the first trial: F(1,28) = 8.263, p = 0.0076]. nNOS KO mice traveled a greater distance [Fig 2D–F; genotype effect, first trial: F(1,28) = 7.901, p = 0.0089, a day after the first trial: F(1,28) = 6.266, p = 0.0184, 7 days after the first trial: F(1,28) = 7.816, p = 0.0093], reflecting the hyperactivity in the nNOS KO mice. In the second and third trials, immobility during the first minute was similar to that during the last minute in the previous trial in wild-type mice [immobility in last minute of the first trial vs. immobility in first minute of the second trial, t(16) = 1.679, p = 0.1126; immobility in last minute of the second trial vs. immobility in first minute of the third trial, t(16) = 0.500, p = 0.6240, paired-t test], suggesting that wild-type mice remembered the previous event. On the other hand, nNOS KO mice showed less immobility during the first 1 min in the second or third trials compared to that during the last minute in the previous trial [immobility during the last minute of the first trial vs. immobility during the first minute of the second trial, t(12) = 3.442, p = 0.0049; immobility during the last minute of the second trial vs. immobility during the first minute of the third trial, t(12) = 3.875, p = 0.0022, paired-t test]. This finding might indicate that nNOS KO mice have impaired reference memory for stressful events.

Together, these data suggest that nNOS deletion impairs spatial working memory, remote spatial reference memory and reference memory for stressful events.

### Increased D1-mediated dopaminergic signaling in brain slices of nNOS KO mice

**Figure 5:**
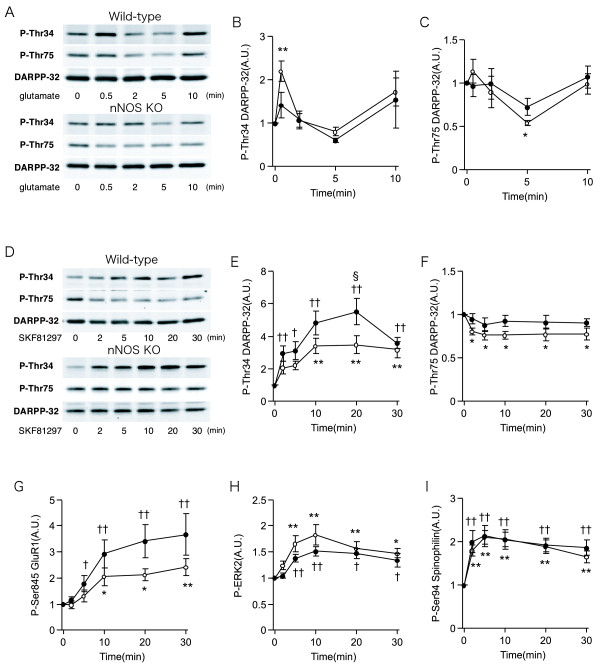
**Regulation of protein phosphorylation by glutamate and a D1 receptor agonist in brain slices from nNOS KO mice**. Neostriatal slices from wild-type (open circles) and nNOS KO (closed circles) mice were treated with glutamate (5 mM) (A-C) and a dopamine D1 receptor agonist, SKF81297 (1 μM; D-I) for the indicated times. Changes in the phosphorylation of DARPP-32 at Thr34 (B, E) and Thr75 (C, F), GluR1 at Ser845 (G), ERK2 (H), and spinophilin at Ser94 (I) were determined by Western blotting using phosphorylation-state specific antibodies. Typical immunoblots detected with phosphorylation-state specific and total DARPP-32 antibodies are shown in (A, D). Data represent means ± SEM for 3–9 experiments. *p < 0.05, **p < 0.01 compared with untreated slices (time 0) from wild-type mice; †p < 0.05, ††p < 0.01 compared with untreated slices (time 0) from nNOS KO mice; one-way ANOVA followed by Newman-Keuls test. §p < 0.05 compared with values of wild-type mice; two-way ANOVA followed by Bonferroni test.

Glutamate and dopamine regulate DARPP-32 phosphorylation in neostriatal neurons via the activation of multiple signaling cascades [45]. To examine the effect of nNOS deletion on glutamatergic and dopaminergic signaling, we investigated the regulation of protein phosphorylation by glutamate and a D1 receptor agonist in striatal slices from nNOS KO mice (Fig 5).

Treatment of neostriatal slices from wild-type mice with glutamate (5 mM) induced a rapid increase in DARPP-32 Thr34 phosphorylation after 30 s incubation, but the effect was transient (Fig 5A, B). We previously reported that the effects of glutamate are mediated via the activation of nNOS/NO/cGMP/PKG signaling [45]. The glutamate-induced increase in Thr34 phosphorylation was absent in nNOS KO mice. Other than the rapid and transient increase in Thr34 phosphorylation, the phosphorylation levels of DARPP-32 at Thr34 and Thr75 under basal conditions and during incubation with glutamate were similar between wild-type and nNOS KO mice [genotype effect on DARPP-32 Thr34, F(1, 31) = 0.4146, p = 0.1927; genotype effect on DARPP-32 Thr75, F(1, 32) = 0.01168, p = 0.6053; Fig 5B,C]. The D1 receptor agonist SKF81297 (Fig 5D–I) increased the phosphorylation of DARPP-32 at Thr34 (Fig 5E), GluR1 at Ser845 (Fig 5G), ERK2 (Fig 5H), and spinophilin at Ser94 (Fig 5I), all of which are phosphorylated by PKA, in both wild-type and nNOS KO mice. The increases in DARPP-32 Thr34 and GluR1 Ser845 phosphorylation were significantly higher in nNOS KO mice than in wild-type mice [genotype effect on DARPP-32 Thr34, F(1, 96) = 12.47, p = 0.0007; genotype effect on GluR1 Ser845, F(1, 94) = 9.199, p = 0.0032], but the increases in ERK2 and spinophilin Ser94 phosphorylation were similar between wild-type and nNOS KO mice [genotype effect on ERK2, F(1, 94) = 2.491, p = 0.1189; genotype effect on spinophilin Ser94, F(1, 96) = 0.6535, p = 0.4212] (Fig 5E,G–I)]. These results suggest that dopamine D1 receptor signaling is upregulated in the striatum of nNOS KO mice in a substrate specific manner.

Treatment of wild-type slices with SKF81297 decreased DARPP-32 Thr75 phosphorylation, presumably via the activation of PP-2A/B56δ by PKA and increased dephosphorylation of Thr75 [57,58] (Fig 5F). The SKF81297-induced decrease in Thr75 phosphorylation was not observed in striatal slices from nNOS KO mice [genotype effect on DARPP-32 Thr75, F(1, 98) = 12.74, p = 0.0006], suggesting that regulation of PP-2A activity by D1 receptor/PKA signaling is also altered in nNOS KO mice.

### Increased D1-mediated dopaminergic signaling in nNOS KO mice in the PPI test

**Figure 6:**
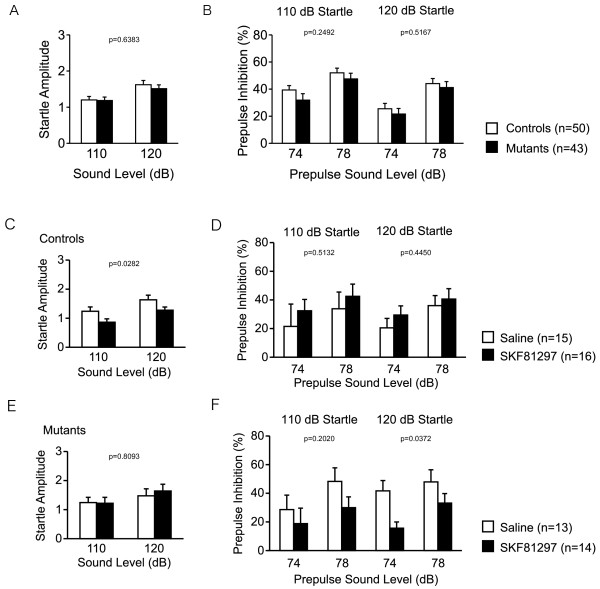
**D1 receptor agonist-induced disruption of prepulse inhibition in nNOS KO mice**. Startle responses at 110 dB and 120 dB were recorded (A). Startle response following 74 dB and 78 dB prepulse inhibition stimuli were recorded (B). Effect of SKF81297 administration on startle responses and prepulse inhibition in wild-type mice (C, D) and in nNOS KO mice (E, F). The p values indicate genotype effect (A, B) and drug effect (C-F) in repeated measures ANOVA.

Though low dose of D1 receptor agonist does not alter PPI in mice [59], high dose of it disrupts PPI [60]. D1 receptor antagonist also disrupted PPI [61,62]. Because dopamine D1 receptor signaling seems to be upregulated in nNOS KO mice, we examined the effect of D1 receptor agonist, SKF81297, on PPI in nNOS KO mice. PPI is a cross-species phenomenon in which the startle response is reduced when the startle stimulus is preceded by a low intensity prepulse, and is disrupted in certain neuropsychiatric disorders that are characterized by abnormal sensorimotor gating, such as schizophrenia [61]. The effect of dopamine agonists on PPI differs between species, and D1 receptor agonists disrupt PPI in mice [62-64]. nNOS KO mice and wild-type mice were tested in the PPI test without drug and with a low dose of the D1 receptor agonist SKF81297 (1 mg/kg, intraperitoneally). In the test without drug, acoustic startle and PPI were not significantly different across genotypes [Fig 6A; genotype effect, F(1,91) = 0.223, p = 0.6383; Fig 6B; genotype effect, F(1,91) = 1.345, p = 0.2492 (110 dB), F(1,91) = 0.424, p = 0.5167 (120 dB)]. Wild-type mice showed a significantly decreased acoustic startle response following injection with SKF81297 [Fig 6C; drug effect, F(1,29) = 5.336, p = 0.0282], but nNOS KO mice did not (Fig 6E). Neither injection with saline nor SKF81297 significantly altered PPI in wild-type mice (Fig 6D). In contrast, PPI was disrupted in nNOS KO mice after injection with SKF81297 [Fig 6F; drug effect, F(1, 25) = 5.115, p = 0.0327 (120 dB)]. These data indicate that PPI is easier to disrupt with a D1 receptor agonist in nNOS KO mice compared to wild-type mice, and suggest that nNOS KO mice have upregulated D1 mediated signaling. Thus, increased D1-mediated dopaminergic signaling was also demonstrated in nNOS KO mice at the behavioral level.

## Discussion

In the present study, nNOS KO mice exhibited increased locomotor activity in the open field test. Hyperactivity of nNOS KO mice was also consistently observed in other tests, such as the light/dark transition, elevated plus maze, and social interaction (novel environment) tests. To date, locomotor activity of nNOS KO mice has not been well examined. Although a few studies have reported on the locomotor activity of nNOS KO mice [24,26,65], most of the observations were either not quantitative and/or subject animals were not compared with appropriate control animals. Nelson et al. reported that nNOS KO mice displayed normal locomotor activity, but they in fact assessed locomotor balance and coordination [24]. In the study by Bilbo et al., nNOS KO mice showed increased locomotor activity in the open field test only in the second test, performed during the dark phase, but not during the light phase [65]. In addition, they compared nNOS KO mice with non-littermate C57BL/6 controls. Comparing behaviors between mutant mice and non-littermate mice may lead to the detection of an effect of different environments rather than the mutation itself. Behavioral phenotyping of genetically engineered mice should be done with control animals from the same standardized genetic and environmental background as the engineered mice [66,67]. Otherwise, an observed difference in the phenotype could be caused by differences in the breeding environment or genetic background. Weitzdoerfer et al. used an observational test battery and reported increased locomotor activity in nNOS KO mice [26]. Because their methods depended heavily on human observation, however, the data were not quantitative. They used nNOS KO mice with only three-generations of backcrossing [26]. In mice from an N3 backcross, there is theoretically 12.5% of the donor strain genome, therefore the phenotype could be caused by flanking genes [68]. The donor strain of the nNOS KO mice in their study was 129/Sv, which is hypoactive compared to the C57BL/6 strain. The hyperactivity of nNOS KO mice may have therefore been masked by the low activity of the donor strain in their study. Mice used in the present study were N5 backcrossed and had a predominantly C57BL/6J genetic background (94.5%), assessed by analyzing 100 microsatellite makers. Because further backcrossing into C57BL/6J leads to low fertility in nNOS KO mice, we could not backcross them further. Gryuko et al. also reported that complete elimination of nNOS, including splicing variants, caused infertility [47].

Pharmacologic studies indicate that administration of a NOS inhibitor reduces locomotor activity in rodents [69-71]. NOS inhibitors also cause a lack of motor coordination in rodents, assessed by the rotarod test [71]. There is a high expression of nNOS in the cerebellum and motor coordination is highly cerebellar-dependent. If administration of an nNOS inhibitor induces an acute lack of motor coordination, this may present as reduced locomotor activity. In our study, although muscle strength, as assessed by the grip strength test, was reduced in nNOS KO mice, they performed normally in the rotarod test, suggesting that other molecule(s) or residual splicing variants of nNOS [47] compensate for the loss of nNOS. With normal motor coordination, nNOS KO mice might display increased locomotor activity. Kriegsfeld et al. reported a deficit in the balance and coordination of nNOS KO mice in a balance test on the pole and plank only during the dark phase [25]. In our study, all experiments except the home cage social interaction test were performed during the light phase, therefore the effect of a lack of motor coordination on locomotor activity in nNOS KO mice, if any, would be smaller than that during the dark phase.

### Abnormal social behavior of nNOS KO mice

Although there are some studies reporting the social behavior of nNOS KO mice [26,72] and the social behavior of animals treated with nNOS inhibitors [72-75], the results are inconsistent. Some studies report that treatment with a NOS inhibitor decreases social interaction behavior [72,75]; others report that treatment with a NOS inhibitor [74] and nNOS knockout [26] does not affect social interaction behavior; and still others report that treatment with a NOS inhibitor increases social interaction behavior [73]. These contradictory findings might be the result of different methods or conditions used to assess social behavior. Additionally, in most of the studies, only one kind of experiment was conducted, and therefore it is difficult to compare the results between them. In the present study, to assess social behavior of nNOS KO mice in various situations, we conducted four kinds of social interaction tests. nNOS KO mice showed 1) an increased number of contacts and an increased total duration of active contacts in a novel environment (one-chamber social interaction test), which might reflect hyperactivity, 2) increased social interaction behavior in their home cage, and 3) decreased social approach behavior in Crawley's three-chamber social approach test.

In the social interaction test in a novel environment (one-chamber social interaction test), the mouse was exposed to a stranger mouse in the chamber and both mice were able to move freely. Weitzdoerfer et al. reported normal social interaction behavior in nNOS KO mice [26]. Their experimental conditions for the social interaction test were similar to those of our one-chamber test. Although the indices they used were the number of social behaviors such as sniffing, grooming, mounting, rubbing, and fighting, all of which are heavily dependent on human observation, their findings were consistent with ours.

The social interaction test in the home cage in the present study revealed increased social interaction behavior in nNOS KO mice. Although there are no reports of increased social interactions in nNOS KO mice, a pharmacologic study showed that the administration of a NOS inhibitor increases social interactions in rats [73]. The finding that NOS inhibition in rats increases social interactions in a novel environment, and nNOS deficiency in mice increases social interactions in familiar environments may reflect an interspecies difference.

In the sociability test (three-chamber), the stranger mouse was in a wire cage and was unable to move freely, and the subject mouse could therefore approach the stranger mouse. Additionally, because this test apparatus (three-chamber) was larger than the one-chamber apparatus, the subject mouse could remain far away from the stranger than in the one-chamber social interaction test. This situation may decrease social investigative behavior in nNOS KO mice. Although nNOS KO mice spent less time around the stranger than the control mice, there was no significant difference between genotypes in the time spent around the empty cage and the distance traveled, indicating that nNOS KO mice showed normal exploration of novel objects and novel environments. Therefore, the decreased social investigative behavior of nNOS KO mice was not due to neophobia.

In the social novelty preference test, a different stranger mouse contained in a wire cage was added to the empty chamber in the sociability test, and then the subject mouse was allowed to explore the cage with the familiar mouse and the cage with the stranger mouse. Distance traveled by wild-type mice in the social novelty preference test was reduced compared to that in the sociability test, probably because those tests were conducted in succession. The ratio between the distance traveled in the social preference test and that in the sociability test was higher in nNOS KO mice than in control mice, indicating that exploration was reduced less in nNOS KO mice than in control mice. Impaired habituation of nNOS KO mice in the three-chamber test may be interpreted as a cognitive impairment. In a study by Bohme et al., rats treated with an NOS inhibitor did not show reduced exploration of a juvenile rat during a second exposure, suggesting that NOS inhibition impaired social recognition [74]. Their finding was consistent with ours in the social novelty preference test. nNOS inhibition also impairs olfactory learning [74], which might be reflected by the impaired habituation of the nNOS KO mice in the present study.

Trainor et al. demonstrated decreased social interaction behavior in nNOS KO mice [72] in which a stranger mouse isolated by a wire barrier was introduced into the home cage and the social behavior of nNOS KO mice was observed. nNOS KO mice spent less time near the barrier compared to control mice, indicating decreased social investigation by the nNOS KO mice. Trainor et al. reported a similar finding in mice administered an nNOS inhibitor. Although their experiments were performed using a home cage, the conditions of the test were similar to those of our three-chamber sociability test regarding the introduction of an animal into the cage. Thus, the results of our three-chamber sociability test are consistent with their findings, i.e., decreased social investigative behavior by nNOS KO mice.

In the present study, nNOS KO mice showed abnormal social behaviors, such as increased social interaction in their home cage, decreased social investigation in a social preference test, and normal social behaviors in the one-chamber social interaction test. Together, the findings from the various social behavior tests indicated that nNOS KO mice demonstrate increased social behavior with a familiar mouse in familiar conditions and they exhibit normal or mildly decreased social behavior with an unfamiliar mouse. Dysregulated social behaviors are often observed in patients with psychiatric disorders such as schizophrenia. Associations between the nNOS gene and schizophrenia have been reported [29,76-78]. Moreover, a recent study demonstrated significantly more disruption or structural variants in genes involved in NO signaling pathways in schizophrenic patients than in normal controls [31], suggesting the involvement of nNOS and NO signaling pathways in schizophrenia. Thus, abnormal nNOS function might be involved in dysregulated social behavior in a subpopulation of schizophrenic patients.

### Impaired reference memory retention and working memory in nNOS KO mice

The eight-arm radial maze task is a hippocampus-dependent task that is generally used to evaluate working memory in rodents [79,80]. Both nNOS KO and wild-type mice exhibited normal working memory when the delay between each arm choice was 5 s. nNOS KO mice exhibited mild deficits in working memory, however, when the delay was increased to 30 s.

Spatial reference memory is frequently assessed by the hidden platform version of the Morris water maze, another test dependent on hippocampal function [81]. In a previous study, nNOS KO mice showed deficits in reaching the hidden platform 10 to 14 days after training in the Morris water maze; although memory immediately after training was not examined, the findings were interpreted as a memory recall deficit [26]. In the same study, nNOS KO mice performed well in a multiple T-maze test, a less stressful spatial task, leading the authors to conclude that the memory deficit of the nNOS KO mice was observed only under stressful conditions [26]. In the present study, however, both nNOS KO and wild-type mice spent significantly more time in the targeted quadrant than in the other three quadrants 1 day after the last training (day 15), indicating that the nNOS KO mice are able to learn, remember, and recall the platform location normally with short retention delays, even under stressful conditions. Further, these findings indicate that the spatial memory deficit of the nNOS KO mice is not likely due to an abnormal sensitivity to stress. On the other hand, nNOS KO mice failed to search the target quadrant 7 days after the last training (day 23), suggesting that the nNOS KO mice have impaired memory retention rather than impaired memory recall. These results are consistent with an idea that NO acts as an important retrograde message for long-term potentiation (LTP) [13] In hippocampal slices of nNOS KO mice, NO-dependent LTP was only slightly reduced, but otherwise normal, probably because the lack of NO was compensated for by the endothelial isoform of NO (eNOS) [82-84] or by residual nNOS splice variants [85]. A recent study revealed a potential role of nNOS in late-phase LTP [86], a finding that is consistent with the behavior of nNOS KO mice in the water maze test in the present study, because late-phase LTP involvement is implicated in the maintenance/storage of long-term memory [87].

### Increased D1 receptor-mediated protein phosphorylation in nNOS KO mice

Activation of dopamine D1 receptors stimulates cAMP/PKA signaling, leading to the phosphorylation of PKA substrates such as DARPP-32 at Thr34 and GluR1 at Ser845 in striatal neurons [37]. Phosphorylation of DARPP-32 and GluR1 induced by the activation of dopamine D1 receptors was enhanced in nNOS KO mice compared to wild-type mice. However, enhanced D1 receptor/PKA signaling was not detected in the analysis of spinophilin Ser94 and ERK2 phosphorylation. The biochemical study clearly demonstrated that D1 receptor/PKA signaling in nNOS KO mice is upregulated in striatal neurons, although the upregulation is substrate-specific. The increase in D1 receptor/PKA signaling detected in the striatum of nNOS KO mice could be applied for brain regions involved in PPI, and supports the findings of D1 receptor-mediated disruption of PPI in nNOS KO mice. In addition, activation of the D1 receptors is known to induce an increase in locomotor activity [88] and a decrease in depression-related behavior in the forced swim test [89]. Hyperactivity and a decrease in depression-related behavior in Porsolt forced swim test, observed in nNOS KO mice, might be explained by the upregulated D1 receptor signaling.

As nNOS deletion in somatostatin-positive interneurons and the subsequent reduction of NO/guanylyl cyclase/cGMP/PKG signaling in medium spiny neurons upregulates D1 receptor/PKA signaling in the striatum, it is possible that the NO/PKG pathway has an inhibitory influence on D1 receptor/adenylyl cyclase/cAMP/PKA signaling in medium spiny neurons, leading to the suppression of DARPP-32 Thr34 and GluR1 Ser845 phosphorylation. Thus, the NO/PKG pathway has bidirectional effects on DARPP-32 Thr34 phosphorylation: PKG and PKA phosphorylate DARPP-32 at Thr34, whereas PKG likely inhibits D1 receptor/adenylyl cyclase/cAMP/PKA signaling upstream of DARPP-32. The molecular mechanisms by which PKG modifies D1 receptor/adenylyl cyclase/cAMP/PKA signaling require further elucidation.

Activation of dopamine D1 receptors decreases the phosphorylation of DARPP-32 at Thr75 (Cdk5-site) via PKA-dependent activation of PP-2A/B56δ [57,58]. In agreement, DARPP-32 Thr75 phosphorylation was decreased by D1 receptor activation in wild-type mice. In contrast, nNOS KO mice did not show any changes in DARPP-32 Thr75 phosphorylation after D1 receptor activation. The findings are different from the predicted results, because PKA signaling is upregulated and PKG signaling, which increases DARPP-32 Thr75 phosphorylation [90], is downregulated in nNOS KO mice. The reason for the lack of Thr75 dephosphorylation in response to D1 receptor activation in nNOS KO mice is unknown. Phospho-Thr75 DARPP-32 inhibits PKA, and the inhibition is removed when D1 receptor/PKA signaling is activated [57]. The positive feedback loop for PKA activation seems to be impaired in nNOS KO mice possibly due to the high PKA tone. Alternatively, it is possible that activity of PP-2A/B56δ is modulated by PKG, although highly speculative.

PPI of an acoustic startle induces a reduced startle response to the startle stimulus when the stimulus is immediately preceded by a weaker prestimulus [91]. PPI is naturally observed in humans and other animals including rodents, but it is often disrupted in psychiatric disorders such as schizophrenia [92]. PPI is disrupted by pharmacologic manipulations with a psychotomimetic drug, phencyclidine, and dopamine agonists [61]. The role of nNOS in the behavioral effect of phencyclidine has been investigated using the NOS inhibitor and nNOS KO mice. A NOS inhibitor, L-NAME, blocked the phencyclidine-induced decrease in PPI [93,94]. Interestingly, the effect of phencyclidine in nNOS KO mice was opposite [95]. In that study, treatment with phencyclidine increased PPI in nNOS KO mice but not in wild-type control mice [95]. Although there is a discrepancy between studies using the NOS inhibitor and nNOS KO mice, those studies demonstrate that nNOS/NO signaling plays a critical role in the regulation of PPI.

In the present study, activation of D1 receptors with a low dose of SKF81297 disrupted PPI in nNOS KO mice, but not in wild-type mice. A number of studies demonstrated that manipulations of dopamine signaling alter PPI in rodents [59-62,64], and pharmacologic studies indicate the relationship between high dopamine signaling and disruption of PPI [61]. SKF81297, a D1 agonist, in a relatively high dose compared to that used in the present study, decreased PPI [60], whereas the D1 antagonist increased PPI in rats [96,97]. It is likely that dopamine D1 receptor signaling is upregulated in nNOS KO mice as demonstrated by biochemical studies, and therefore PPI is disrupted in nNOS KO mice in response to a low dose of SKF81297.

There are several studies that report abnormal behavior of hyperdopaminergic mice. Dopamine transporter (DAT) knockdown mice, that are known as hyperdopaminergic, displayed hyperactivity [98,99], perseverative motor behavior [99], and impaired response habituation [98]. In addition, DAT knockout mice also showed hyperactivity, perseverative motor behavior, disrupted prepulse inhibition, and high sensitivity to D1 receptor antagonist [100]. Behavioral phenotypes observed in nNOS KO mice, such as hyperactivity, perseverative motor behavior (increased stereotypic behavior in open filed test), impaired habituation in three-chamber social interaction test, and hypersensitivity to D1 receptor antagonist in PPI test, resemble those in DAT knockdown and knockout mice, suggesting the hyperdopaminergic state of nNOS KO mice.

## Conclusion

nNOS KO mice were subjected to a battery of behavioral tests. nNOS KO mice exhibited hyperactivity, impaired memory, decreased depression-related behavior, abnormal social behavior and D1 receptor-mediated disruption of PPI. Biochemical analysis in the striatum revealed the upregulation of dopamine D1 receptor/PKA signaling in nNOS KO mice. Some of behavioral abnormalities in nNOS KO mice such as hyperactivity, decreased depression-related behavior and D1 receptor-mediated disruption of PPI might be explained by high activity of dopamine D1 receptor/PKA signaling.

## Competing interests

The authors declare that they have no competing interests.

## Authors' contributions

TM and AN are responsible for the original concept and overall design of the research. KTanda, KTakao, KN, NY, KToyama, and TM performed the behavioral analysis of mutant mice. AN performed biochemical assays. KTanda, KTakao, NM, TS, AN and TM wrote the manuscript. All authors read and approved the final manuscript.
